# Per2 Upregulation in Circulating Hematopoietic Progenitor Cells During Chronic HIV Infection

**DOI:** 10.3389/fcimb.2020.00362

**Published:** 2020-07-21

**Authors:** Veronica Bordoni, Eleonora Tartaglia, Giulia Refolo, Alessandra Sacchi, Germana Grassi, Andrea Antinori, Gian Maria Fimia, Chiara Agrati

**Affiliations:** ^1^Laboratory of Cellular Immunology, National Institute for Infectious Diseases “L. Spallanzani” IRCCS, Rome, Italy; ^2^Laboratory of Cell Biology and Electron Microscopy, National Institute for Infectious Diseases “L. Spallanzani” IRCCS, Rome, Italy; ^3^Clinical Department, National Institute for Infectious Diseases “L. Spallanzani” IRCCS, Rome, Italy; ^4^Department of Molecular Medicine, University of Rome “Sapienza”, Rome, Italy

**Keywords:** hematopoietic progenitor cells, HIV, senescence, telomere length, period circadian clock 2, Sirtuin 1

## Abstract

Chronic HIV infection accelerates immune aging and is associated with abnormal hemato-lymphopoiesis, but the relationship between HIV-induced aging and Hematopoietic Progenitor Cells (HPC) function is not well-defined. In the context of aging, it has been demonstrated using a murine model that Per2 (Period circadian clock 2) is a negative regulator of HPC survival and lineage potential. A possible involvement of Per2 modulation on hematopoietic failure during HIV infection has not yet been investigated. The aim of this study was to analyze whether Per2 is differently expressed and regulated on HPC during HIV infection, possibly providing a therapeutic target to restore lymphoid potential in the HPC compartment. To this purpose, Per2 expression in circulating HPC was compared in 69 chronic HIV infected patients under successful ART and in matched 30 uninfected healthy donors (HD). HPC aging was assessed by measuring relative telomere length (RTL), and HPC functionality was evaluated by Colony Forming Cell (CFC) assay from both *ex vivo* HIV+ patients and *in vitro* Per2 overexpressing donors. Our results showed a lower RTL in HPC and a decrease of white progenitor colonies from HIV+ patients with lower CD4 respect to those with higher CD4 T cell count (<500 respect to >500 CD4 T cell/mmc). Interestingly, we found that the frequency of Per2-expressing HPC is higher in HIV+ patients than in HD and correlated to RTL of CFC derived cells, highlighting a relationship between low proliferative rate and Per2 expression. Indeed, the *in vitro* overexpression of Per2 resulted in a significant decrease of white progenitor colonies respect to control cells. Finally, we showed that the deacetylase Sirtuin 1, a negative regulator of Per2, was downregulated in HPC from HIV+ patients, and the peripheral blood treatment with resveratrol (Sirtuin 1 inducer) determined a decrease of Per2 expressing HPC. Altogether, these results suggest that during HIV infection, Per2 is involved in the regulation of HPC expansion and differentiation and its overexpression may impair the immune reconstitution. These data support the rationale to explore the role of this regulatory mechanism during aged-associated hemato-lymphopoiesis impairment in HIV infection.

## Introduction

It is generally accepted that stem cell aging is the primary factor driving the aging of tissues characterized by high cell turnover such as the immune system (Rossi et al., 2008; Geiger et al., 2013). Indeed, hematopoiesis declines with age, resulting in a reduced production of immune cells (a process termed immune senescence) and in an increased frequency of myeloid malignancies (Beerman et al., 2010; Pietras et al., 2011). Aged hematopoietic progenitors cells (HPC) exhibit an impairment in lymphoid and erythroid lineage differentiation, while maintain or increase myeloid lineage differentiation potential (Geiger et al., 2013). This decline is thought to contribute to the evolution of immune defects, limiting overall fitness, and organismal survival during aging (Su et al., 2013). A robust indicator of the proliferative history of a cell, and how close this cell is to reaching senescence, somehow reflecting its “age,” is represented by telomere length (Hodes et al., 2002). Indeed, telomeres (long nucleotide repeats at the end of the chromosomes) shorten with every cell division, and thus are markers for cellular aging, senescence, and replicative capacity.

Even if life span is getting closer to that of the general population, HIV+ patients, and mainly the older ones, present an increased prevalence of age-related comorbidities (Pathai et al., 2014; Lagathu et al., 2017). An important matter is whether aging mechanisms associated with HIV-infection are similar or not to those observed in the general population. In HIV infected subjects the immune senescence has been associated with negative immune outcomes, such as thymic involution and poor antigen responsiveness (Deeks, 2011; Lagathu et al., 2017). Moreover, the leukocytes telomere length was lower in viremic or ART-controlled HIV-infected patients than in uninfected individuals (Effros, 2000; Zanet et al., 2014; Liu et al., 2015) and was associated with poor immune recovery.

In a murine model of aging, it has been demonstrated that Per2 (circadian rhythm gene) is a negative regulator of HPC survival and lineage potential, suggesting a strict relationship between Per2 expression and hematopoiesis effectiveness (Wang J. et al., 2016). Per2 is a transcription factor that binds E-boxes and is mainly studied in the mammalian central nervous system in the context of circadian rhythms. However, E-boxes have been demonstrated to play a crucial role in lymphopoiesis (Ephrussi et al., 1985) and Wang J. et al. (2016) identified Per2 as a gene regulating HSC potential in the context of critically short telomeres. Moreover, several studies reported that Per2 is regulated at multiple layers by Sirtuin 1, a protein deacetylase that negatively regulates Per2 expression in aging process (Asher et al., 2008; Chang and Guarente, 2013; Wang R. H. et al., 2016). Moreover, Sirtuin 1 is considered a multitask molecule as it may act on co-activators, transcription factors, and signaling molecules (p53), forkhead box O1 (FOXO, and RelA/p65) (Rajendrasozhan et al., 2009) with anti-apoptotic, anti-aging, and anti-inflammatory properties (Rahman et al., 2012; Hwang et al., 2013; Mendes et al., 2017; Chadha et al., 2019). Interestingly, Kwon et al. (2008) reported that HIV transactivator Tat inhibits the Sirt1 deacetylase, resulting in increased acetylation of NF-kB and subsequently in T cell hyperactivation.

Whether Per2 induction in HPC could be involved in hematopoietic failure during HIV infection and the possible Sirtuin 1 implication in this process have not been investigated yet. The aim of this study was to analyze Per2 expression in circulating HPC during chronic HIV infection and its relationship with HIV-associated aging.

## Materials and Methods

### Study Population

The study was performed on 69 peripheral blood samples from HIV+ patients and 36 from healthy individuals (HD) afferent to National Institute of Infectious Diseases (INMI) “Lazzaro Spallanzani” (Rome, Italy). Sixty-nine HIV+ patients on antiretroviral therapy with a CD4 T cell counts ranging from 130 to 2,001 cells/mm^3^ (median 471 cells/mm^3^), and the viral loads undetected at least from 1 year, were recruited for the purpose of this study. All patients were selected by excluding any co-morbidity as HCV, HBV, EBV, MTB co-infections, and malignancies. The HIV-seronegative donors were used as a control and were processed under the same conditions used for samples obtained from HIV-infected donors. The study was approved by the Institutional Review board of INMI “Lazzaro Spallanzani” (Approval No. 70 dated December 17, 2018). Residual blood samples obtained from CD4 and CD8 routine analysis were completely anonymized. The characteristics of the study population are presented in Table 1.

**Table 1:** **Characteristics of the study population.**.

**Parameter**	**HIV+ CD4>500 subset** **(*n* = 33)**	**HIV+ CD4 <500 subset** **(*n* = 36)**	**HD** **(*n* = 36)**
Age (year)	44 (38–49.5)	46 (38.5–50.75)	43 (39–56)
Sex (F/M)	14/19	12/24	21/15
CD4 T cell count	857 (753.5–1,175)	374 (296–430)	
CD4/CD8 T cell ratio	1.3 (0.76–1.86)	0.5 (0.33–0.86)	

All data are presented as median (IQR).

### Peripheral Blood Mononuclear Cells Isolation and CD34+ Cells Purification

Peripheral blood mononuclear cells (PBMCs) were isolated from peripheral blood by density gradient centrifugation (Lympholyte-H; Cedarlane). Freshly isolated CD34+ Hematopoietic Progenitor Cells were purified from PBMC by a two-step magnetic procedure involving a lineage depletion, followed by a CD34 isolation (CD34 Diamond isolation kit, Miltenyi Biotec). For RTL quantification, CD34+Lin- cells were purified from pooled samples and verified the purity by flow cytometry (>85%).

### Reagents and Flow Cytometry

The levels of *ex vivo* Per2 expression was evaluated using whole blood 24 h after draw. Two hundred microliter of whole blood was stained for 20 min at 4°C with surface antibody anti-CD34 APC and anti-CD45 V500 (Becton-Dickinson BD); following monoclonal staining, BD Pharm Lyse (BD Biosciences) lyse solution was used to lyse red blood cells according to standard procedures and fixed with 4% paraformaldehyde. After red cell lysing, cells were incubated with anti-PER2 antibody (ab179813 Abcam) or anti-Sirt1 Alexa Fluor 488 (ab196368 Abcam) for intracellular staining 20 min at room temperature using a permeabilizing buffer containing 0.5% saponin (PBS1X /BSA 1%/NaN3 0.1%/Saponin 0.5%) and then washed with 0.1% saponin buffer. Alexa Fluor 488-coniugated goat anti rabbit (Invitrogen) diluted in staining buffer containing 0.1% saponin was used as secondary antibody. To evaluate cell viability, we used the 7AAD staining. In some experiments 1 mL of whole blood of HIV infected patients was stimulated with different concentrations of Resveratrol (R5010- Sigma-Aldrich) and YK-3-237 (Tocris) overnight at room temperature (see section results for details). After stimulation, the frequency of HPC expressing Per2 was evaluated by flow cytometry. At least 300,000 total cell events were acquired on FACSCanto II (Becton-Dickinson BD) flow cytometer and analyzed using DIVA software.

### DNA Extraction and RTL Quantification

Genomic DNA was extracted from PBMCs with Quick-gDNA miniprep Kit (Zymo Research) according to manufacturer's instruction. The relative telomere length was measured using the Absolute Human Telomere Length Quantification qPCR Assay Kit (AHTLQ; ScienCell Research Laboratories) according to manufacturer's instruction. Briefly, quantitative real-time PCR (qRT-PCR) was performed with 10 ng of total DNA, FastStart Essential DNA Green Master (Roche Life Science), and two primer set, telomere specific primer set (TEL) and single copy reference primer set (SCR). qRT-PCR reactions were always performed in duplicates. The relative telomere length was calculated using the Comparative ΔΔCq (Quantification Cycle Value) method. The value of ΔΔCq was calculated by the difference of ΔCq (TEL) and ΔCq (SCR), the relative telomere length was calculated by 2-ΔΔCq. The run was performed on Corbett Research Rotor-Gene RG-6000.

### Colony-Forming Cell (CFC) Assay

PBMC from subjects were not purified according to CD34 cell expression because of a limited concentration of cells available (Redd et al., 2007). PBMC were seeded at 10^6^ cells/mL into complete methylcellulose medium (MethoCult complete medium with necessary cytokines and growth factors; StemCell Technologies) following the manufacturer's instructions. Each experiment was performed in duplicate (35 mm Petri dish format) and after 14 days of incubation, erythroid colonies (BFU-E, CFU-E) and white colonies (GM-CFU, GEMM-CFU) were counted using an inverted light microscope. Colonies obtained from Methylcellulose Assay (CFC assay) of HIV and HD were selected, washed twice in PBS and pooled for real time PCR analysis of RTL.

### Sirt1 Expression

Total RNA was extracted from PBMC after whole blood Resveratrol stimulation using TRIzol reagent (Thermo Fisher Scientific), and cDNA synthesis was generated from 1 μg of RNA using the reverse transcription kit (Promega) according to manufacturer's recommendations. Real time PCR reactions were performed with the Corbett Research Rotor Gene 6000 analyzer using FastStart Essential DNA Green Master (Roche Life Science) according to the manufacturer's instructions. Two microliter of cDNA was used as template and cycling parameters were 95°C for 10 min, followed by 40 cycles of 92°C 10 s, 62°C 10 s, 72°C 10s. Levels of RNAs were normalized to the GAPDH level using the equation 2^−ΔCt^. Primers Sirt1 forward: F: GCAGATTAGTAGGCGGCTTG, Sirt1 reverse: TCTGGCATGTCCCACTATCA; GAPDH_F: GTGGAATCATATTGGAACATGT GAPDH_R: CTCTCTGCTCCTCCTGTTCGACAG.

### Lentiviral Production and Viral Infection

For lentiviral production, HEK293T cells were cotransfected with 10 μg of lentiviral vectors, 2.5 μg pVSV-G plasmid, and 7.5 μg of psPAX2 plasmid using the calcium phosphate method, as previously described (Refolo et al., 2019). Per2 lentiviral vector specific for human Per2 (pLenti-GIII-CMV-C-term-HA, LV260156) and GFP lentiviral vector used as a control of infection (pLenti-CMV-GFP-2A-Puro) were purchased from ABMGood. Lentiviral productions were tested by infecting HuH7 cells with serial dilutions and counting the number of cells resistant to puromycin selection upon 3 days. In addition, they were tested for the levels of Per2 expression upon 293T infection by western blotting by using anti-HA antibody. 1 × 10^6^ freshly isolated PBMC were infected with 100 μl of viral suspension in RPMI medium supplemented with 4 μg/ml polybrene for 18 h and after collected for flow cytometry and CFC assay.

### Statistics

Quantitative variables were compared with non-parametric Mann–Whitney or Kruskal-Wallis test. Spearman rank test was used to determine correlations. Wilcoxon test was used for matched samples. A *p* < 0.05 was considered statistically significant. Statistical analyses were performed using GraphPad Prism v7.0 (GraphPad Software, Inc.).

## Results

### Telomere Length and Differentiation Capability of HPC From HIV-Infected Patients

To assess the relationship between aging and immune reconstitution during chronic HIV infection, we first evaluated the level of aging and functional properties of circulating Hematopoietic Progenitor Cells (HPC) in comparison to those in age-matched uninfected adult. To this purpose, the non-viremic ART-treated HIV infected patients were separated into two distinct groups according to their CD4 T-cell count, as a marker of progression state and immunological reconstitution: (i) low immunological responder (CD4 T cells <500/mmc, LowIR); (ii) immunological responder (CD4 T cells >500/mmc, IR).

**Figure 1:**
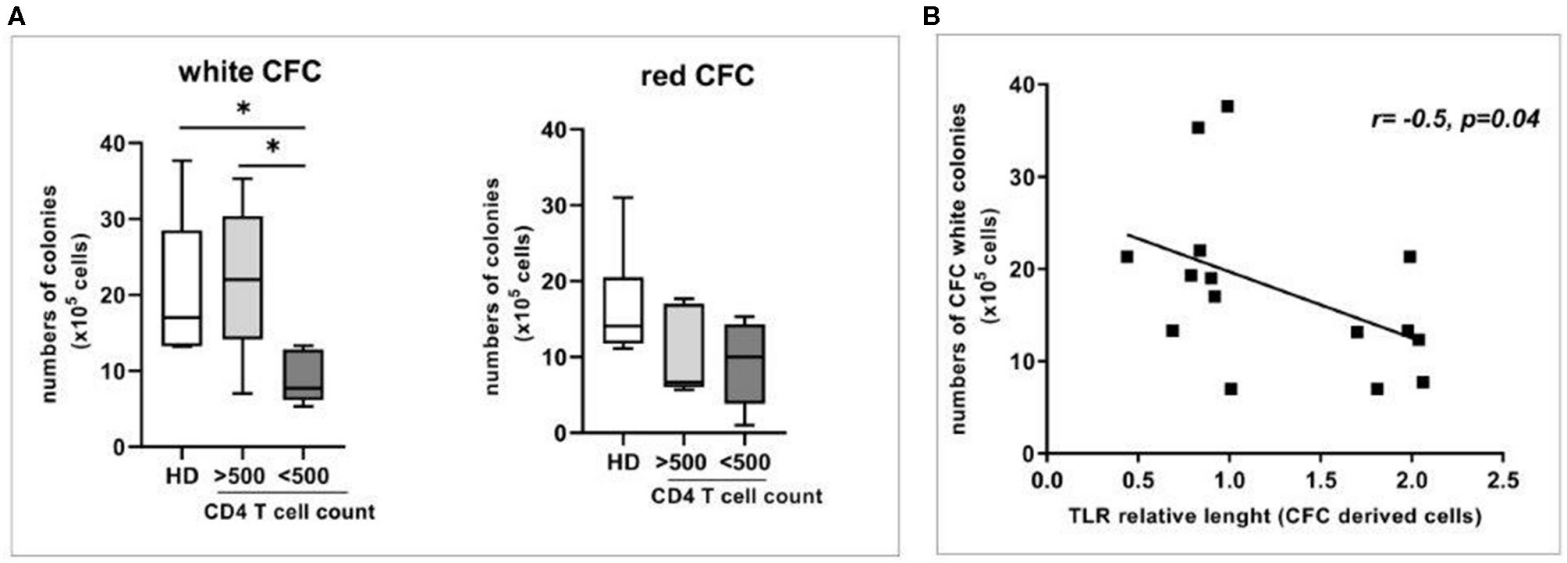
Relative telomere length (RTL) and expansion/differentiation of circulating Hematopoietic Progenitor Cells (HPC) from HD, and HIV-infected patients (<500 and >500 CD4 T cell count). **(A)** Colony forming cell assay (CFC) showing the numbers of white and red colonies (left and right panel) generated from circulating HPC from HD (*n* = 8) and from HIV-infected patients (*n* = 15). **(B)** Correlation between relative telomere length and numbers of CFC white colonies in HD and HIV-infected subjects. Mann–Whitney test, *p < 0.05.

To evaluate a possible association between the immune reconstitution in HIV patients and the expansion/differentiation of HPC, we performed the Colony-Forming Cell (CFC) assay. Specifically, the generation of white [i.e., granulocytes-macrophages (CFU-GM) and granulocyte-erythroid-macrophage-megakaryocyte (CFU-GEMM)] or red (i.e., erythroid CFU and erythroid burst-forming unit) progenitor colonies was analyzed in LowIR and in IR and in HD. We showed that the capacity of circulating HPC to produce white progenitor colonies appeared to be preferentially impaired during HIV disease progression (LowIR vs. IR, *p* < 0.05, and LowIR vs. HD, *p* < 0.05, Figure 1A left panel) in comparison to HD and IR, while the erythroid lineage was not significantly affected (Figure 1A right panel).

To evaluate the impact of HPC senescence, the analysis of relative telomere length (RTL) was performed after CFC assay both in HD and in HIV subjects (Figure 1B). Interestingly, we found an inverse correlation between the telomere length after culture and the number of white CFC (*r*: −0.52, *p* = 0.04), suggesting a block in telomere shortening in patients with an impairment in white cells differentiation.

No impact of different cART regimens (NRTI, nucleoside reverse-transcriptase inhibitors; PI, protease inhibitors; NNRTI, non-nucleoside reverse-transcriptase inhibitors; INSTI, Integrase Strand Transfer Inhibitor) on CD34 frequency was observed [(NRTI+PI: median 0.031%, IQR: 0.025–0.10) vs. (NRTI+NNRTI: median 0.053%, IQR: 0.03–0.08) vs. (NRTI+INSTI: median 0.037, IQR 0.03–0.2)]. Altogether the results highlight a relationship between HPC senescence and dysfunction in ART-treated HIV-infected patients.

### Increase in the Frequency of HPC Expressing Per2 During Chronic HIV Infection

**Figure 2:**
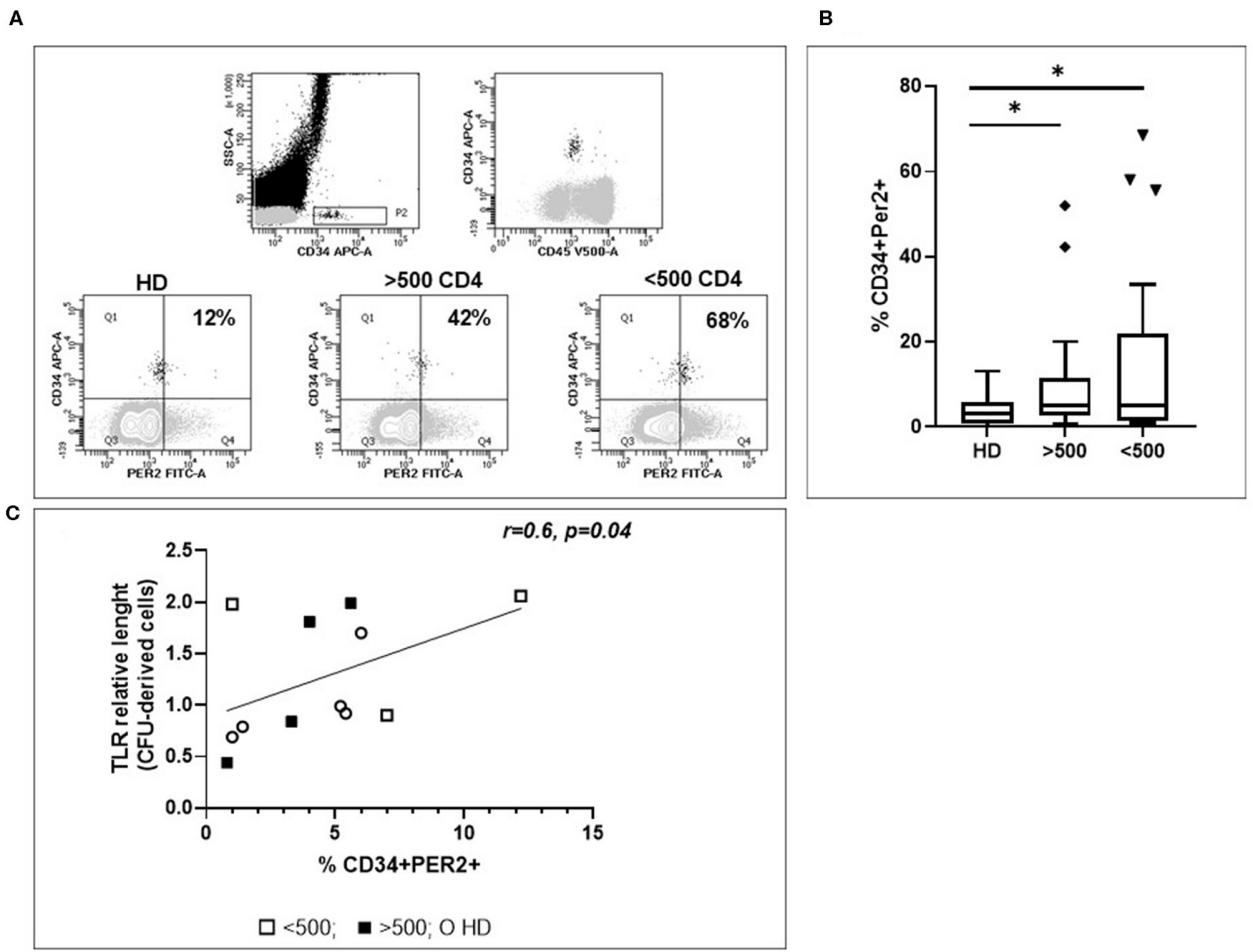
Evaluation of Per2 expression in circulating HPC. **(A)** Representative flow cytometry contour plots of CD34+CD45+/low cells from whole blood of HIV+ patients. In the square the percentage of CD34+Per2+ cells is shown. To set Per-2 positive/negative gate, we used a mix of antibodies containing CD34–APC and the secondary antibody Alexa 488 without Per2 primary antibody. **(B)** Histograms show the median and range percentage of CD34+Per2+ cells of HD and HIV-infected patients. Mann–Whitney test, *p < 0.05; HD, healthy donors (*n* = 32), HIV + patients with CD4 T cells count <500 (*n* = 27), HIV + patients with CD4 T cells count >500 (*n* = 26). **(C)** Linear regression analysis between RTL of CFC derived cells and % CD34+Per2+ cells (*r* = 0.6, *p* = 0.04).

To verify whether the hematopoietic failure observed in HIV infected patients may be associated to a higher Per2 expression, we analyzed the percentage of HPC expressing Per2 in chronically HIV+ patients. Interestingly, we found that the frequency of Per2-expressing HPC is higher in HIV+ patients than in HD [median lowIR = 5% (IQR 1.4–22), median IR = 5% (IQR = 2.7–11.4) vs. median HD = 3% (IQR 0.75–5.25) *p* < 0.05, Figures 2A,B]. No statistically differences in Per2 expression in HPC between lowIR e IR were found.

To assess a possible relationship between Per2 expression and HPC aging, we analyzed the RTL after HPC expansion and differentiation from HIV+ patients. Interestingly, we found that the frequency of Per2-expressing HPC is directly correlated to RTL of CFC derived cells, underlining a low proliferative rate in relationship with Per2 expression (*r* = 0.6, *p* = 0.04, Figure 2C).

### Per2 Overexpression Induces a Decrease in CFC White Colonies Expansion

**Figure 3:**
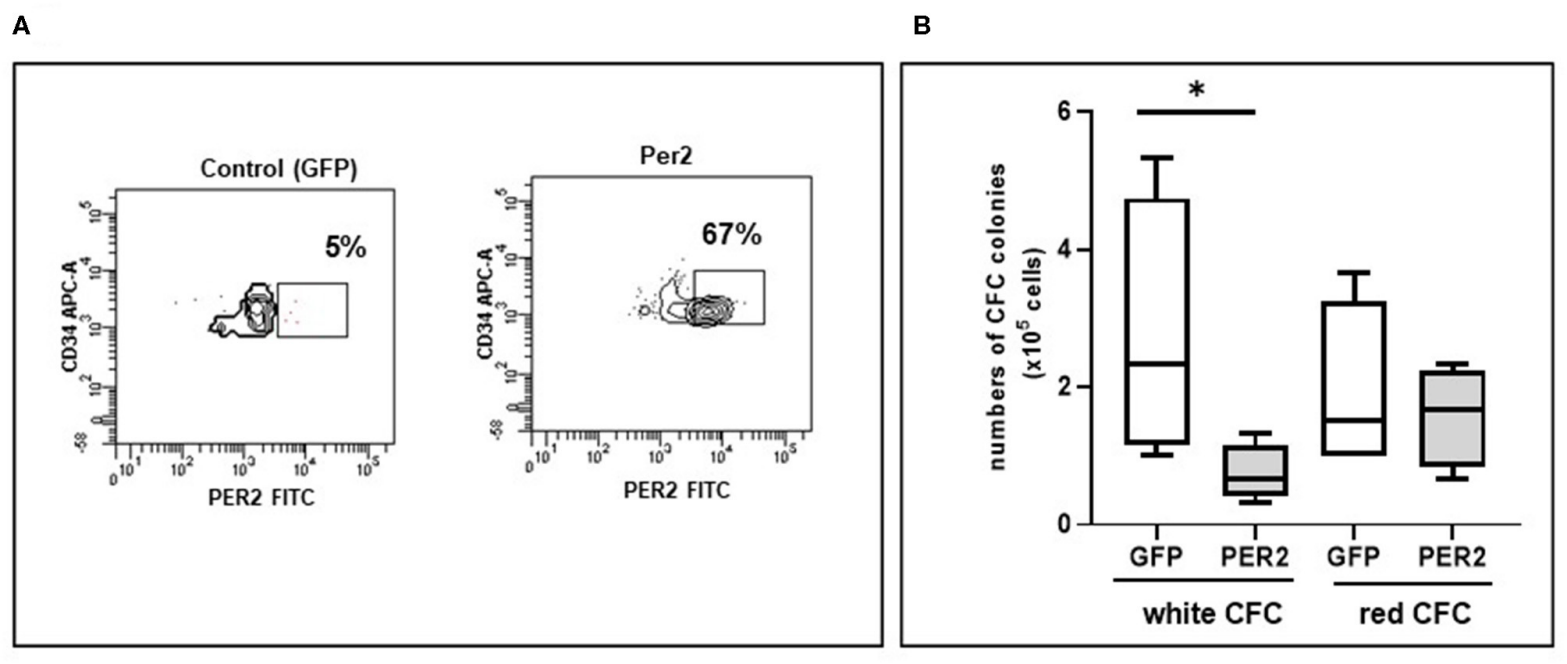
Circulating Per2 expressing-HPC differentiation features. **(A)** Representative flow cytometry contour plots of Per2 expression in HPC after lentiviral infection: in the square is indicated the percentage of CD34+Per2+ cells obtained after infection of PBMC with control lentiviral vector (GFP, left panel) or with Per2 specific lentiviral vector (right panel). **(B)** Colony Forming Cell assay (CFC) showing the absolute value of white and red colonies generated from circulating HPC of HD infected with Per2 specific lentiviral vector or control GFP lentiviral vector. Mann-Whitney test, *p < 0.05.

The above data indicated that Per2 is higher in HIV-infected subjects under successful ART. To finally demonstrate that Per2 is directly associate with the impairment in circulating HPC expansion and differentiation, we overexpressed Per2 in PBMC from HD and performed CFC assay. The lentiviral infection did not affect CD34 frequency. When PBMC were infected with Per2 expressing lentiviral vector, the percentage of CD34+ cells expressing Per2 increase over 60% (Figure 3). We found a significant decrease of white colonies, but not red colonies, when Per2 is overexpressed with respect to GFP infection that was used as control (*p* < 0.05, Figure 3B).

### Sirt1 Expression on HPC and Downregulation of the Expression of Per2 in Response to Resveratrol Stimulation

**Figure 4:**
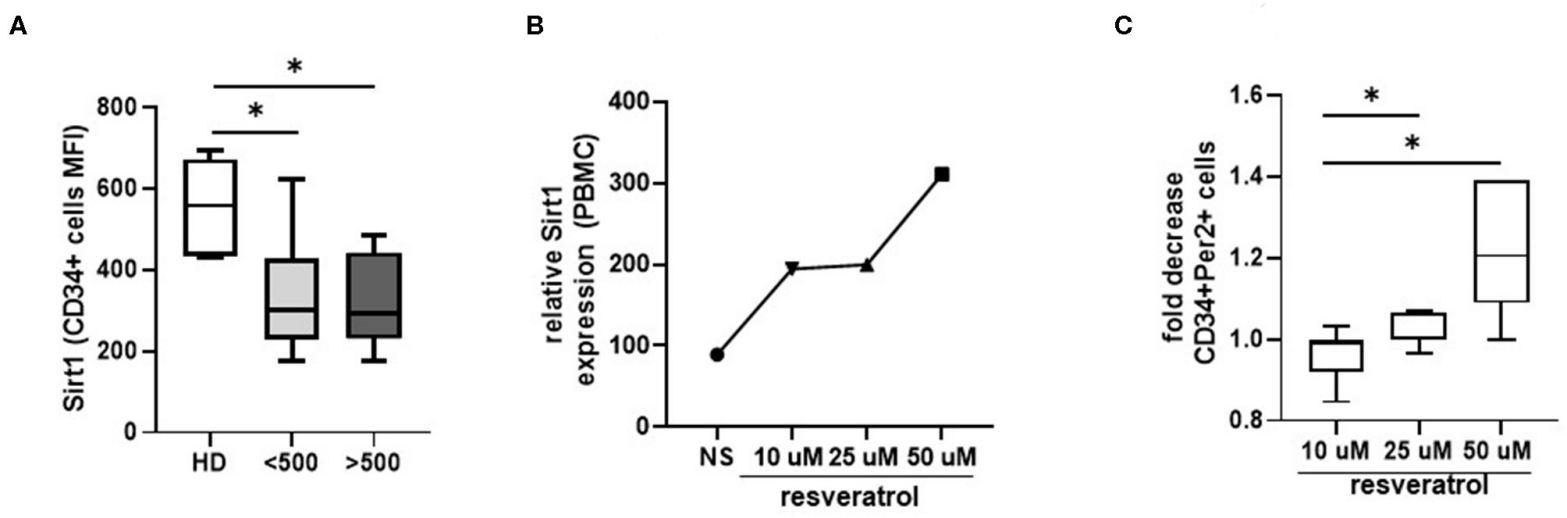
Sirt1 modulation. **(A)** Histograms show the median and range of Sirt1 MFI in CD34+ cells from HD and HIV-infected patients. Mann–Whitney test, *p < 0.05; HD, healthy donors (*n* = 7), <500, HIV+ patients with CD4 T cells count <500 (*n* = 8), >500, HIV+ patients with CD4 T cells count >500 (*n* = 7). **(B)** Relative Sirt1 expression after 24 h of whole blood stimulation with resveratrol (50, 25, and 10 μM concentration). The data refer to the mean of 2 pool (each pool consists of 4 samples). **(C)** Histograms show the median and range of the fold of decrease of CD34+Per2+ cells percentage after 24 h of whole blood stimulation with resveratrol (50, 25, and 10 μM concentration). Wilcoxon matched-pairs signed rank test, *p < 0.05.

To investigate how Per2 is regulated in HPC from HIV+ patients, we analyze the expression of the deacetylase Sirtuin 1 (Sirt1), a negative regulator of Per2 previously associated to aging modulation (Chang and Guarente, 2013). As showed in Figure 4A, we observed a significant lower expression of Sirt1 on circulating HPC from HIV+ patients respect to HD (*p* < 0.05), suggesting a dysregulation in the Per2/Sirt1 pathway during HIV infection. To better elucidate the Per2-Sirt1 axis, we wondered whether the stimulation of Sirt1 expression could modulate the percentage of Per2-expressing HPC. To this purpose, we treated whole blood of HIV+ patients with resveratrol, a compound known to induce the expression and activity of Sirt1, and the percentage of CD34+Per2+ cells was analyzed by flow cytometry. This treatment did not affect the cell viability as measured by 7AAD staining (% 7–AAD+ cells: unstimulated 7.2% vs. Resveratrol 9.8%, *p* = 0.5). As reported in Figures 4B,C, we found that resveratrol induces Sirt1-mRNA in PBMC from HIV+ patients and a significant dose dependent decrease of Per2-expressing HPC (*p* < 0.05). Finally, we tested the efficacy of YK-3-237, a more specific Sirt-1 targeting drug, to reduce Per2 expression on CD34 cells [fold of decrease: YK-3-237 0.01 μM: 0.99 (0.97–1.02) vs. 1 μM:1.04 (1.02–1.07, *p* < 0.03)], confirming the involvement of the Sirt1 in the regulation of Per2 in HPC during chronic HIV infection.

## Discussion

In HIV infected patients, several mechanisms influencing the immune restoration has been described, such as inflammation and immune activation (Sauce et al., 2011; Bordoni et al., 2017), upregulation of apoptosis of CD34+ cells (Isgro et al., 2008), and the reduction of CD34 + CD7 + CXCR4 + T cell progenitors (Tsukamoto, 2019), but the relationship between aging and HPC in ART-treated HIV patients has not been clarified.

Wang and co-authors identifies period circadian clock 2 (Per2) as a critical factor limiting the maintenance of hematopoietic stem cells in response to DNA damage and aging in mice (Wang J. et al., 2016). The authors show that the selective induction of Per2 in lymphoid precursors is likely to contribute to the reduction of lymphopoiesis, and in the maintenance of immune functions in aging mice. In this paper we found that Per2 is overexpressed in circulating HPC of ART treated HIV infected patients respect to healthy donors. Moreover, Per2 expression on HPC is inversely correlated to telomeres length of progenitors, indicating the low replicative potential or growth arrest when Per2 is induced.

In human hepatocytes it was reported that Per 2 expression is negatively regulated by the Sirt1 deacetylase (Wang R. H. et al., 2016). In this study, we found a lower expression of Sirtuin 1 in circulating HPC from HIV patients when compared to healthy controls, suggesting a possible relationship between the low Sirtuin expression and the high Per2 expression on HPC from HIV infected patients. Accordingly, when inducing *in vitro* Sirtuin expression by treating whole blood with resveratrol, the percentage of Per2 expressing HPC significantly decrease. These data showed for the first time the axis sirtuin1-Per2 in HPC in HIV-infected patients and highlight new interesting pathways regulating HPC potential.

A number of *in vivo* studies that utilize Sirt1^−/−^ mice have demonstrated that Sirt1 positively regulates stemness in HSCs, highlighting the involvement of ROS elimination, FOXO activation, and inhibition of p53 (Matsui et al., 2012; O'Callaghan and Vassilopoulos, 2017). However, the involvement Sirt1 down regulation in HPC function during HIV infection has not been elucidated. Furthermore, some sirtuins seem to be involved in aging and cell senescence, although data from different experimental models are controversial (Lin et al., 2000; Mostoslavsky et al., 2006; Rodriguez et al., 2013).

An important role of Sirtuin family has been described in the regulation of inflammatory response (Hwang et al., 2013; Mendes et al., 2017; Chadha et al., 2019). Sirt1 has been shown to suppress NF-κb activity, the regulator of cellular inflammatory response and increase antioxidant gene expression that suppressed inflammation (Mendes et al., 2017). During senescence, the level of Sirt1 mRNA is finely regulated by HuR (Hu antigen R) and Checkpoint kinase (Chk). Decreased levels of HuR and increased levels of CHk2 synergize to decrease Sirt1 mRNA and protein levels during senescence (Kwon and Ott, 2008). Therefore, it is possible that the mechanism of senescence triggered by immune-activation and inflammation lead to a decrease of Sirt1 protein levels.

Finally, Sirt1 can be also inhibited by HIV–Tat protein resulting in increased acetylation of the NF-kB p65 subunit and subsequently in T cell hyperactivation (Kwon et al., 2008). Although this mechanisms can play a role in viremic phase of HIV infection, the patients enrolled in this study were under successful ART (undetectable virus in the circulating blood). Therefore, is unlikely that Sirt1 activity in HPC was inhibited by HIV–Tat protein. Nevertheless, the effect of *in vitro* HIV−1 infection (X4- and R5-tropic strains) on Sirt-1 expression and function could be useful to depict the role of viral components on Sirt-1/Per2 axis.

Our results showed an altered differentiation capacity of circulating HPC from ART-treated HIV infected patients, in line with previous observations obtained in bone marrow samples from viremic HIV subjects (Moses et al., 1998; Sauce et al., 2011). The HPC impairment was associated to a block in telomere shortening after long term culture, suggesting a relationship between HPC senescence and functional impairment in HIV disease. Of note, we showed that the overexpression of Per2 may modulate HPC expansion and differentiation, as confirmed by *in vitro* overexpression. Further analysis are mandatory to understand the factors driving Sirt-1/Per2 dysregulation observed in HIV infected patients and their possible impact on lymphopoiesis and immune reconstitution efficacy. Moreover, we have to take into account a possible involvement of different drugs of ART regimens on Per2 expression. In fact, it was reported that the ART regimens containing the nucleoside analog reverse transcriptase inhibitors can alter telomerase reverse transcriptase activity, resulting in telomere length shortening in leukocytes (Hukezalie et al., 2012; Leeansyah et al., 2013; Lagathu et al., 2017).

Altogether, these results suggest that in viral suppressed HIV-infected patients the axis Sirt1/Per2 is unbalanced: Per2 increase in HPC and its overexpression may contribute to the impairment in immune reconstitution and accelerate the aging process.

## Data Availability Statement

The datasets generated for this study are available on request to the corresponding author.

## Ethics Statement

This study was approved by the Institutional Review board of INMI Lazzaro Spallanzani (Approval No. 70 dated December 17, 2018). Written informed consent for participation was not required for this study in accordance with the national legislation and the institutional requirements.

## Author Contributions

VB conceived the study, performed the experiments, analyzed the data, and prepared the manuscript. AA was charge of patients. ET performed the experiments and analyzed the data. AS analyzed the data and provided comments to the manuscript. GR performed the lentiviral infection experiments. GG performed flow cytometry experiments. CA and GF analyzed the data and prepared the manuscript. All authors read, reviewed, and approved the final manuscript.

## Conflict of Interest

The authors declare that the research was conducted in the absence of any commercial or financial relationships that could be construed as a potential conflict of interest.
